# Impaired amygdala astrocytic signaling worsens neuropathic pain-associated neuronal functions and behaviors

**DOI:** 10.3389/fphar.2024.1368634

**Published:** 2024-03-21

**Authors:** Mariacristina Mazzitelli, Olga Ponomareva, Peyton Presto, Julia John, Volker Neugebauer

**Affiliations:** ^1^ Department of Pharmacology and Neuroscience, School of Medicine, Texas Tech University Health Sciences Center, Lubbock, TX, United States; ^2^ Center of Excellence for Translational Neuroscience and Therapeutics, Texas Tech University Health Sciences Center, Lubbock, TX, United States; ^3^ Garrison Institute on Aging, Texas Tech University Health Sciences Center, Lubbock, TX, United States

**Keywords:** amygdala, astrocyte, neuroimmune signaling, electrophysiology, behavior, neuronal excitability, neurotransmission, neuropathic pain

## Abstract

**Introduction:** Pain is a clinically relevant health care issue with limited therapeutic options, creating the need for new and improved analgesic strategies. The amygdala is a limbic brain region critically involved in the regulation of emotional-affective components of pain and in pain modulation. The central nucleus of amygdala (CeA) serves major output functions and receives nociceptive information via the external lateral parabrachial nucleus (PB). While amygdala neuroplasticity has been linked causally to pain behaviors, non-neuronal pain mechanisms in this region remain to be explored. As an essential part of the neuroimmune system, astrocytes that represent about 40–50% of glia cells within the central nervous system, are required for physiological neuronal functions, but their role in the amygdala remains to be determined for pain conditions. In this study, we measured time-specific astrocyte activation in the CeA in a neuropathic pain model (spinal nerve ligation, SNL) and assessed the effects of astrocyte inhibition on amygdala neuroplasticity and pain-like behaviors in the pain condition.

**Methods and Results:** Glial fibrillary acidic protein (GFAP, astrocytic marker) immunoreactivity and mRNA expression were increased at the chronic (4 weeks post-SNL), but not acute (1 week post-SNL), stage of neuropathic pain. In order to determine the contribution of astrocytes to amygdala pain-mechanisms, we used fluorocitric acid (FCA), a selective inhibitor of astrocyte metabolism. Whole-cell patch-clamp recordings were performed from neurons in the laterocapsular division of the CeA (CeLC) obtained from chronic neuropathic rats. Pre-incubation of brain slices with FCA (100 µM, 1 h), increased excitability through altered hyperpolarization-activated current (I_h_) functions, without significantly affecting synaptic responses at the PB-CeLC synapse. Intra-CeA injection of FCA (100 µM) had facilitatory effects on mechanical withdrawal thresholds (von Frey and paw pressure tests) and emotional-affective behaviors (evoked vocalizations), but not on facial grimace score and anxiety-like behaviors (open field test), in chronic neuropathic rats. Selective inhibition of astrocytes by FCA was confirmed with immunohistochemical analyses showing decreased astrocytic GFAP, but not NeuN, signal in the CeA.

**Discussion:** Overall, these results suggest a complex modulation of amygdala pain functions by astrocytes and provide evidence for beneficial functions of astrocytes in CeA in chronic neuropathic pain.

## 1 Introduction

The availability of effective therapeutic options for the treatment of chronic pain remains inadequate and limited, resulting in a major health issue worldwide and the need for a better understanding of pain mechanisms to provide insights into novel treatment approaches (Furlan et al., 2006; Chou et al., 2009; Martel et al., 2015). Evidence from clinical and preclinical studies suggests that the amygdala, a limbic brain structure, is critically involved in the emotional aspect of pain and pain modulation. The central nucleus of amygdala (CeA) serves major pain-related output functions within the amygdala circuitry. The laterocapsular division of CeA (CeLC) receives nociceptive information via the parabrachial (PB) nucleus in the brainstem, and this pathway has been linked to the modulation of pain behaviors (Veinante et al., 2013; Thompson and Neugebauer, 2017; Neugebauer, 2020). Pain-related changes in amygdala activity are now well documented in rodents (Thompson and Neugebauer, 2017; Thompson and Neugebauer, 2019; Neugebauer, 2020) and humans (Simons et al., 2014), but underlying mechanisms are not fully understood. Recent evidence suggests that pain mechanisms cannot be explained solely by neural factors, but alterations of the neuroimmune system, a key player in maintaining homeostasis in the central nervous system (CNS), have been linked to pain induction and maintenance (Grace et al., 2014; Ji et al., 2018; Ji et al., 2019; Donnelly et al., 2020). Astrocytes represent the majority of the glial cells in the CNS and serve critical physiological functions related to neuronal metabolism and synapse processes, but their contribution to the pathophysiology of pain in different brain areas is not clear (Grace et al., 2014; Ji et al., 2014; Ji et al., 2016; Donnelly et al., 2020; Cheng et al., 2022). In pathological conditions, astrocytes become activated (“reactive”), undergoing a series of structural and functional changes that serve to protect the brain (Tian et al., 2012; Ji et al., 2019). A large body of evidence has focused on the contribution of reactive glia to nociceptive processing at the peripheral and spinal cord levels (Li et al., 2019; Miranpuri et al., 2021; Xu et al., 2021; Cheng et al., 2022; Lu and Gao, 2023). Astrocyte activation has also been implicated in several brain regions involved in pain processing (Raghavendra et al., 2004; Narita et al., 2006; Roberts et al., 2009; Chen et al., 2012; Kim et al., 2016; Ni et al., 2019), but the astrocytic contribution to amygdala-related neuroplasticity and behaviors in the context of pain has not been reported. Interestingly, evidence from clinical studies detected higher glial responses at the neuroforamina (dorsal root ganglion and nerve roots) and spinal cord levels (Albrecht et al., 2018) as well as in various brain regions, including thalamus, somatosensory cortex, cingulate cortex and ventromedial prefrontal cortex, in patients suffering from chronic pain (Loggia et al., 2015; Albrecht et al., 2019; Albrecht et al., 2021), supporting the translational relevance of this line of research. Here, we sought to determine the involvement of astrocytes in the right amygdala (CeA) in neuroplasticity and pain behaviors at the chronic (4 weeks) stage of a neuropathic pain model, using a selective astrocytic inhibitor (fluorocitric acid, FCA).

## 2 Materials and methods

### 2.1 Animals

Experimental procedures were approved by the Institutional Animal Care and Use Committee (IACUC; protocol #21026) at Texas Tech University Health Sciences Center and conform to the guidelines of the International Association for the Study of Pain (IASP) and National Institutes of Health (NIH). For patch-clamp experiments, male heterozygous transgenic Crh-Cre(+)/tdTomato(+) rats (Crh, corticotropin releasing hormone also referred to as CRF) on Sprague Dawley background (bred in house) were used to record from non-CRF neurons, which are the predominant cell type in the capsular CeA where the most prominent GFAP signal changes were found in the pain model (see 3.1). For behavioral, immunohistochemical and qRT-PCR assays, male Sprague Dawley rats were purchased from Envigo (Indianapolis, IN). All animals, 250–350 g (2–3 months old) at time of testing, were housed in a temperature-controlled room under a 12 h day/night cycle with unrestricted access to food and water. Rats were randomly assigned to the different experimental groups, and a researcher blinded to drug treatments, and where applicable condition, carried out the experimentation.

### 2.2 Neuropathic pain model

The well-established spinal nerve ligation (SNL) model was used in this study to induce a neuropathy in the left hind paw of rats (Kim and Chung, 1992). Rats were anesthetized with isoflurane (2%–3%; precision vaporizer, Harvard Apparatus) and the L5 spinal nerve was tightly ligated using sterile procedures. Sham operated animals undergoing the same surgical procedure without L5 nerve ligation were used as control group.

### 2.3 Drugs

DL-fluorocitric acid (FCA) barium salt (#F9634) and barium chloride (BaCl_2_, #217565) were purchased from Sigma-Aldrich. Because FCA was commercially available as Ba^2+^ salt, which may interfere with neuronal physiological properties leading to confounding results, we decided (Chou et al., 2009) to use BaCl_2_ salt in control patch-clamp experiments and (Furlan et al., 2006) to precipitate Ba^2+^ during drug preparation for *in vivo* stereotaxic microinjections. For electrophysiology experiments (see 2.6), BaCl_2_ was dissolved in H_2_O and FCA in HCl 0.1 M as a stock solutions and diluted (1:100) to the final concentration (100 µM) in artificial cerebrospinal fluid (ACSF, in mM: 117 NaCl, 4.7 KCl, 1.2 NaH_2_PO_4_, 2.5 CaCl_2_, 1.2 MgCl_2_, 25 NaHCO_3_ and 11 glucose) on the day of the experiment (Khan et al., 2019; Vizuete et al., 2019). For intra-CeA microinjection (see 2.7.1), the compound was dissolved in HCl 0.1 M and the Ba^2+^ was precipitated by the addition of (2–3 drops) Na_2_SO_4_ 0.1 M. This solution was buffered with Na_2_HPO_4_ 0.1 M and centrifuged at 800 *g* for 10 min. The supernatant was removed and diluted in ACSF to the final concentration (100 µM) and pH was adjusted to 7.3 (Paulsen et al., 1987; Hayakawa et al., 2010; Paquette et al., 2021).

### 2.4 qRT-PCR

At either the acute phase (1 week post-SNL or sham surgery) or chronic phase (4 weeks post-SNL or sham surgery) of neuropathic pain (see 2.2), rats were euthanized by decapitation. Brains were rapidly extracted and oxygenated in an ice-cold sucrose-based physiological solution of the following composition (in mM): 87 NaCl, 75 sucrose, 25 glucose, 5 KCl, 21 MgCl_2_, 0.5 CaCl_2_, and 1.25 NaH_2_PO_4_. Two coronal brain slices (1,000 μM) containing the amygdala were prepared using a Vibratome (VT1200S, Leica Biosystems, Nussloch, Germany) as described previously (Presto and Neugebauer, 2022; Presto et al., 2023). The right CeA was dissected for mRNA analysis. RNA was extracted using the MagMAX-96 Total RNA Isolation Kit (Life Technologies, Carlsbad, CA, United States) and quantified on a NanoDrop 8000 spectrophotometer (Thermo Fisher Scientific, Rockford, IL, United States). Total RNA was reverse transcribed using the High-Capacity cDNA Reverse Transcription Kit with RNase Inhibitor (Thermo Fisher Scientific). Taqman Fast Advanced Master Mix (Thermo Fisher Scientific) was used to perform quantitative reverse transcription polymerase chain reactions (qRT-PCR). Applied Biosystems Taqman Gene Expression Assays included GFAP (Gfap; Rn01460868_m1), β-actin (Actb; Rn00667869_m1), ribosomal protein L3 (Rpl3; Rn01505100_g1), and ribosomal protein L29 (Rpl29; Rn00820801_g1). Reactions containing 5 ng of cDNA were conducted in duplicate using the CFX384 Real-Time System (BioRad, Hercules, CA, United States). Relative mRNA expression was determined using the 2^−ΔΔCt^ method, standardizing samples to the geometric mean of β -actin, Rpl3, and Rpl29; these reference genes have been shown to exhibit consistent expression in a rat neuropathic pain model (Wan et al., 2010) and serve as reliable internal markers for the analysis of mRNA expression within the CeA tissue of neuropathic rats in our prior studies (Presto and Neugebauer, 2022; Presto et al., 2023).

### 2.5 Immunohistochemistry

Rats were deeply anesthetized (4% isoflurane; precision vaporizer, Harvard Apparatus) and transcardially perfused with phosphate buffer saline (PBS) (0.01 M phosphate, 0.0027 M KCl and 0.137 M NaCl, pH ∼7.4), followed by 4% paraformaldehyde in PBS (PFA). Brains were extracted and fixed overnight in 4% PFA, cryoprotected for 48 h in 30% sucrose in PBS, frozen in OCT and sectioned at 40 µm thickness using a cryostat (Epredia HM525 NX, Epredia, Kalamazoo, MI, United States). Similar brain sections containing the amygdala area were selected for immunostaining experiments. Sections were permeabilized in 0.3% Triton X100 in PBS (PBST) for 10 min, blocked in 5% normal goat serum (NGS) in PBS for 1 h and incubated with primary antibodies overnight at 4°C. The following day sections were washed with PBS, incubated with secondary antibodies for 2 h at room temperature, and finally mounted using ProLong Mountant (Invitrogen, P36961) after washes with PBS. Primary antibodies used: chicken anti-GFAP (Novus, NBP1-05198, 1:1,000), mouse anti GFAP-Alexa488 conjugated (Invitrogen, eBioscience 53-9892-80, 1:500) and guinea pig anti-NeuN (Millipore, ABN90, 1:1,000). Secondary antibodies used: goat anti-chicken Alexa488 (Invitrogen, A11039, 1:1,000) and goat anti-guinea pig Alexa 647 (Invitrogen, A21450, 1:1,000). Sections were imaged at the identical conditions with a confocal microscope (Olympus FV3000), using a 7 × 7 mosaic taken with ×60 oil immersion objective. Image analysis was performed with ImageJ software (ImageJ v1.53f51, NIH). The different amygdala regions were identified according to the Paxinos Watson Rat Brain Atlas (Paxinos and Watson, 2006). After marking amygdala regions in the NeuN channel, capsular and lateral divisions of CeA (CeC and CeL) were filled with sample areas of 250 × 250 px using “Rectangle” tool while avoiding large blood vessels. For GFAP expression, images were thresholded in the GFAP channel, and in each sample area the GFAP positive (+) area was analyzed using Analyze Particles function. In each section, multiple sample areas were analyzed (CeC, 21–39; CeL, 9–20) and averaged. Each group contained three animals with 3-4 sections per animal. For the effect of FCA injections on GFAP and NeuN positive signals, mosaic images taken with ×10 objective in two channels (GFAP and NeuN) were analyzed. Amygdala regions were marked in the NeuN channel. Images were thresholded in the analyzed channels (GFAP or NeuN), new region of interest (ROI) was created while avoiding large blood vessels, and percent of signal positive area or mean of positive signal were measured using Analyze Particles function. Each group had five animals, and one section including same region (CeL and CeC) per animal was evaluated.

### 2.6 Patch-clamp electrophysiology

Brain slices containing the right amygdala were obtained from neuropathic rats 4 weeks after SNL surgery as described previously (Kiritoshi and Neugebauer, 2018; Hein et al., 2021; Mazzitelli et al., 2022; Yakhnitsa et al., 2022), because significant changes of astrocytic marker GFAP were found only at the chronic neuropathic stage (see 3.1). Brains were quickly removed and immersed in an oxygenated ice-cold sucrose-based physiological solution (see 2.4). Coronal brain slices (400 μm) were obtained using a Vibratome (Leica Biosystems VT Series, Buffalo Grove, IL) and incubated in oxygenated ACSF (see 2.3) at room temperature (21°C). Slices were incubated with FCA barium salt or control solutions (BaCl_2_, 100 μM, in ACSF; or ACSF alone; see 2.3) for 1 h before recordings. Then, an individual brain slice was transferred to the recording chamber and submerged in ACSF (31°C ± 1°C) superfusing the slice at ∼2 mL/min. Residual Ba^2+^ salt due to the pre-treatment was quickly washed out by ACSF in the recording chamber. Only two brain slices per animal were used, and 2–3 neurons were recorded in each brain slice. Whole-cell patch-clamp recordings were made from visually identified non-CRF neurons in the lateral-capsular division of the CeA (CeLC) using DIC-IR videomicroscopy and fluorescence illumination (BX51, Olympus, Waltham, MA) as described previously (Kiritoshi et al., 2013; Kiritoshi and Neugebauer, 2018; Mazzitelli et al., 2022; Yakhnitsa et al., 2022). Recording pipettes (tip resistance 5–8 MΩ) were made from borosilicate glass and filled with an intracellular solution containing (in mM): 122 K-gluconate, 5 NaCl, 0.3 CaCl_2_, 2 MgCl_2_, 1 EGTA, 10 HEPES, 5 Na_2_-ATP, and 0.4 Na_3_-GTP; pH was adjusted to 7.2–7.3 with KOH and osmolarity to 280 mOsm/kg with sucrose. Data acquisition and analysis were done using a dual four-pole Bessel filter (Warner Instr., Hamden, CT), low-noise Digidata 1550B interface (Axon Instr., Molecular Devices, Sunnyvale, CA), MulitClamp700B amplifier (Axon Instr., Molecular Devices, Sunnyvale, CA), and pClamp11 software (Axon Instr.). If series resistance (monitored with pClamp11 software) changed more than 20%, the neuron was excluded from analysis. To characterize the frequency-current (F–I) relationship as a measure of excitability, depolarizing current pulses (500 ms, 25 pA step) were applied. The total number of action potentials generated was measured at each current magnitude, and the rheobase was calculated as the smallest current step that elicited an action potential. Additionally, the firing threshold was identified by applying a single ramp voltage protocol. The depolarizing voltage sag was measured by the application of hyperpolarizing currents (500 ms, 20 pA steps) and was calculated as the difference between the largest decrease in voltage and the steady state value (ΔV). Current-voltage (I-V) relationship was obtained by voltage clamping the neuron at −60 mV and measuring current amplitudes in response to voltage steps (20 mV, 300 ms). Synaptic responses were evoked in voltage-clamp, using a concentric bipolar stimulating electrode (David Kopf Instruments) to stimulate glutamatergic afferent inputs presumably from the PB as in our previous studies (Kiritoshi and Neugebauer, 2018; Hein et al., 2021; Mazzitelli et al., 2022; Yakhnitsa et al., 2022). Mono-synaptic glutamatergic excitatory (EPSC) and glutamate-driven feedforward inhibitory (IPSC) post-synaptic currents were evoked at −70 mV and 0 mV, respectively. Peak amplitude, area under the curve (AUC), and decay time were analyzed. For paired-pulse ratio (PPR) analysis, two orthodromic synaptic stimuli of equal intensity (50-ms interstimulus interval) were applied and EPSCs were recorded (−70 mV). Peak amplitudes of the first EPSC (EPSC1) and the second EPSC (EPSC2) were measured and PPR was calculated as the ratio of EPSC2 over EPSC1.

### 2.7 Behavioral assays

Behavioral assays were performed 16–24 h after intra-CeA microinjection of FCA or vehicle (see 2.7.1) and 4 weeks after the neuropathic surgery (see 2.2). The following behavioral assays were performed in shielded temperature- and light-controlled rooms. Each rat underwent all behavioral assays in the following order: nocifensive reflex threshold using von Frey test (see 2.7.2), spontaneous behavior (facial grimace scoring) (see 2.7.3), anxiety-like behavior (see 2.7.4), emotional-affective behavior (evoked vocalizations) (see 2.7.5), and nocifensive reflex threshold using a calibrated forceps (see 2.7.2). The evoked behavior tests (reflexes and vocalizations) were repeated twice in the same animal and then averaged.

#### 2.7.1 Intra-CeA microinjection

To inhibit astrocytes in the CeA, FCA was injected into the right CeA of SNL rats (see 2.2) using a stereotaxic apparatus (David Kopf Instruments, Tujunga, CA, United States) as previously described (Hein et al., 2021; Mazzitelli et al., 2022; Presto et al., 2023). Animals were anesthetized with isoflurane (2%–3%; precision vaporizer, Harvard Apparatus, Holliston, MA, United States) and a small unilateral craniotomy was performed. FCA (100 μM) or vehicle (Ba^2+^ was removed by precipitation; see 2.3) was injected (1 μL, 10 min) with a 5 μL Hamilton syringe using the following coordinates: 2.5 mm caudal to bregma, 4.0–4.3 mm lateral to midline, and 7.3–7.6 mm deep.

#### 2.7.2 Mechanosensitivity

Nocifensive reflex thresholds were assessed using a plantar electronic von Frey anesthesiometer (IITC Life Science, Woodland Hills, CA) or a calibrated forceps connected to a force transducer whose output was displayed in grams on an LCD screen to gradually compress the hind paw with continuously increasing intensities (paw compression test); the force required for evoking a reflex response was displayed on an LCD screen and recorded. Von Frey tests were performed before (baseline) and 4 weeks after SNL surgery (see 2.2) to confirm the mechanical allodynia induced by the neuropathic pain model. To determine the effects of the intra-CeA treatment on mechanosensitivity, von Frey or paw pressure tests were performed on both left (injured) and right hind paws 16–24 h after drug delivery in neuropathic rats (see 2.7.1).

#### 2.7.3 Spontaneous behaviors

Facial rodent grimace scale (RGS) was used to assess spontaneous pain behaviors (Sotocinal et al., 2011). The rats were placed in individual plexiglass chambers with home cage bedding in a quiet environment. A video camera (Sony Handycam HDR-CX455 9.2 megapixels with lenses Zeiss Vario-Tessar, Sony Corporation of America, New York, NY, United States) was placed on the outside of the chambers and each rat was video-recorded for a 5-min period. Ten images (at least 30 s apart) of the rat face were randomly selected from the still images using a random number generator. Only those images were selected that showed rats directly facing the camera; for images that did not qualify for analysis, a new random number was generated. Three final images for scoring were then selected using a random number generator and assigned a random number code. Scoring was performed by five treatment-blinded experienced evaluators (Sotocinal et al., 2011). Each image was scored based on four action units: orbital tightening, nose/cheek flattening, ear changes and whisker change. A score from 0 to 2 (0 = not present, 1 = moderate, 2 = severe) was assigned to each facial unit (parameter). Scores were entered in an excel spreadsheet. The four action unit scores were summed to produce the total score and a mean of the scores for all three images was obtained.

#### 2.7.4 Anxiety-like behaviors

The open field test (OFT) was used to investigate anxiety-like behavior in SNL rats (see 2.2). Behavioral activity in a square arena (70 cm × 70 cm) was videotracked for 5 min with EthoVision (Noldus Information Technology, Leesburg, VA, United States). Time spent (s) in the center area of the field (35 cm × 35 cm) and locomotor activity as the total distance traveled (cm) were calculated for the first 5 min. Avoidance of the center area of the OFT was interpreted as anxiety-like behavior.

#### 2.7.5 Emotional responses

Vocalizations in the audible (20 Hz–16 kHz) and ultrasonic (25 ± 4 kHz) ranges were measured in neuropathic rats (see 2.2) as described before (Brudzynski, 2007; Neugebauer et al., 2007; Thompson et al., 2015; Mazzitelli and Neugebauer, 2019; Presto et al., 2021). Rats were briefly anesthetized with isoflurane (2%–3%, precision vaporizer) to minimize stress of handling, and placed in a custom-designed recording chamber (U.S. Patent 7,213,538) to ensure a fixed distance from the sound detectors. A microphone connected to a preamplifier was used to record audible vocalizations, and a bat detector connected to a filter and amplifier measured ultrasonic vocalizations. (UltraVox four-channel system; Noldus Information Technology). After recovery from the brief anesthesia, vocalizations were evoked by brief (10 s) normally innocuous (300–500 g/6 mm^2^), and noxious (1,000–1,500 g/6 mm^2^) stimuli applied to the left (injured) or right hind paws using a calibrated forceps (see 2.7.2). Vocalizations were recorded for 1 min and analyzed using Ultravox 2.0 software (Noldus Information Technology) as described before (Neugebauer et al., 2007; Presto et al., 2021; Mazzitelli et al., 2022; Yakhnitsa et al., 2022). Innocuous stimulation preceded noxious stimulation.

### 2.8 Data and statistical analysis

All averaged values are presented as means ± SEM. GraphPad Prism 10.0 software (Graph-Pad Software, San Diego, CA) was used for all statistical analyses. Statistical significance was accepted at the level *p* < 0.05. Two-way ANOVA (repeated measures as appropriate) with Bonferroni posthoc tests or one-way ANOVA with Tukey’s posthoc tests were used for multiple comparisons, and unpaired t-tests were used for comparison of two sets of data that had Gaussian distribution and similar variance as indicated.

## 3 Results

### 3.1 Increased astrocyte activation at the chronic but not acute stage of neuropathic pain

**FIGURE 1:**
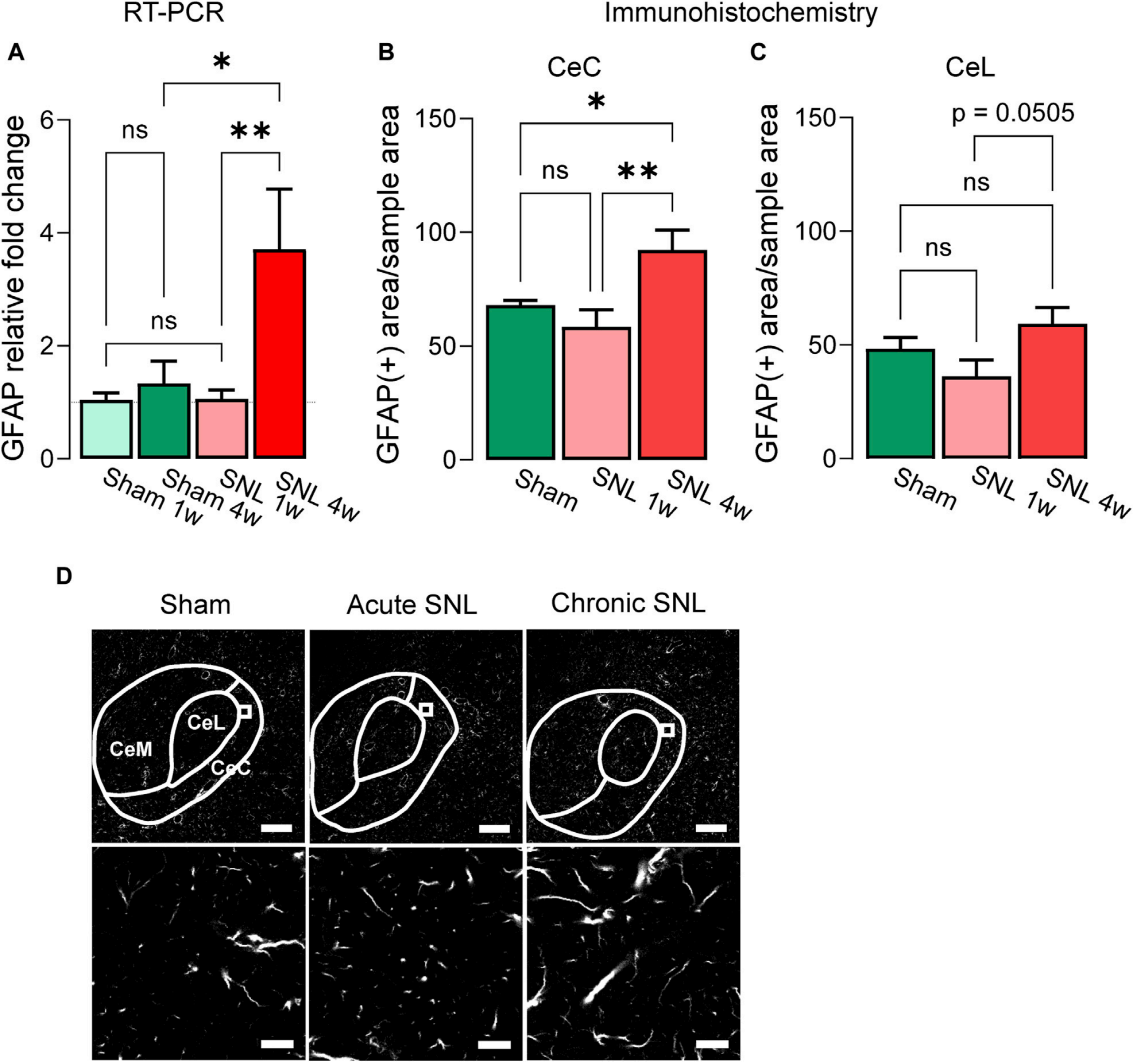
Temporal changes of GFAP mRNA and protein in the CeA of neuropathic rats. **(A)** Increased GFAP mRNA expression levels in the right CeA 4 weeks, but not 1 week, post-induction of SNL compared to sham control group. Importantly, no significant differences were observed between the two sham control groups (sham 1w vs. sham 4w). Bar histograms show means ± SEM. *,**p < 0.05, 0.01, two-way ANOVA with Bonferroni posthoc tests. Sham 1w, n = 7; sham 4w, n = 4; SNL 1w, n = 7; SNL 4w, n = 4. **(B)** Immunohistochemistry shows significantly increased GFAP (+) signal in CeC of chronic SNL rats compared to acute SNL or sham groups. A similar trend was detected in the CeL **(C)** though no statistical significance was reached (*p* = 0.0505). **(D)** Representative images of GFAP (+) signal in the CeA of different treatment groups. Bottom: representative sample area used for analysis. Scale bars, top, 200 μm; bottom, 10 μm *, **, *p* < 0.05, 0.01, one-way ANOVA Tukey’s posthoc tests. CeC and CeL: sham, n = 10, SNL 1w, n = 10, SNL 4w, n = 10 sections from three animals per group.

Levels of GFAP mRNA expression in the right CeA were examined at the acute (1 week post-SNL or sham surgery) or chronic (4 weeks post-SNL or sham surgery) stages of neuropathic pain (Figure 1A). RT-PCR analysis showed significant differences in the GFAP mRNA expression level between the acute and chronic time points and compared to sham control animals (sham 1w, n = 7; sham 4w, n = 4; SNL 1w n = 7; SNL 4w, n = 4; treatment (column) factor, F_(3, 12)_ = 6.618, *p* = 0.0069; Subject (row) factor, F_(6, 12)_ = 0.6736, *p* = 0.6737, two-way ANOVA). Specifically, GFAP mRNA expression in the CeA of SNL rats was increased compared to sham rats at the chronic (sham 4w, n = 4; SNL 4w, n = 4) but not acute (sham 1w, n = 7; SNL 1w n = 7) stage of neuropathic pain (*p* < 0.05, two-way ANOVA with Bonferroni’s *post hoc* tests). GFAP mRNA expression levels were significantly higher at the chronic compared to the acute stage of SNL (*p* < 0.01, two-way ANOVA with Bonferroni’s *post hoc* tests), suggesting a delayed upregulation of astrocytic mRNA in the CeA in neuropathic pain. No significant differences in GFAP mRNA expression levels were found between the acute and chronic sham cohorts; therefore, 4-week sham rats were used as control for the subsequent experiments.

Immunohistochemical analysis showed that the GFAP (+) signal was significantly enhanced in the CeC of chronic SNL rats compared to acute SNL or sham groups (Figures 1B, D, sham = 10, SNL 1w = 10, SNL 4w = 10 total GFAP(+) sample areas per section, 3-4 sections from three animals per group; F_(2,27)_ = 6.477; *p* = 0.0050, one-way ANOVA with Tukey’s multiple comparison tests). In the CeL, a clear trend (*p* = 0.0505, one-way ANOVA with Tukey’s posthoc tests) for increased GFAP (+) signals at chronic SNL compared to acute SNL was observed, but there were no statistically significant differences between the three groups (Figure 1C, sham = 10, SNL 1w = 10, SNL 4w = 10 total GFAP (+) sample areas per section, 3-4 sections from three animals per group; F_(2,27)_ = 3.065; *p* = 0.0631, one-way ANOVA with Tukey’s posthoc tests).

### 3.2 Electrophysiological effects of astrocyte inhibition at the chronic stage of neuropathic pain

**FIGURE 2:**
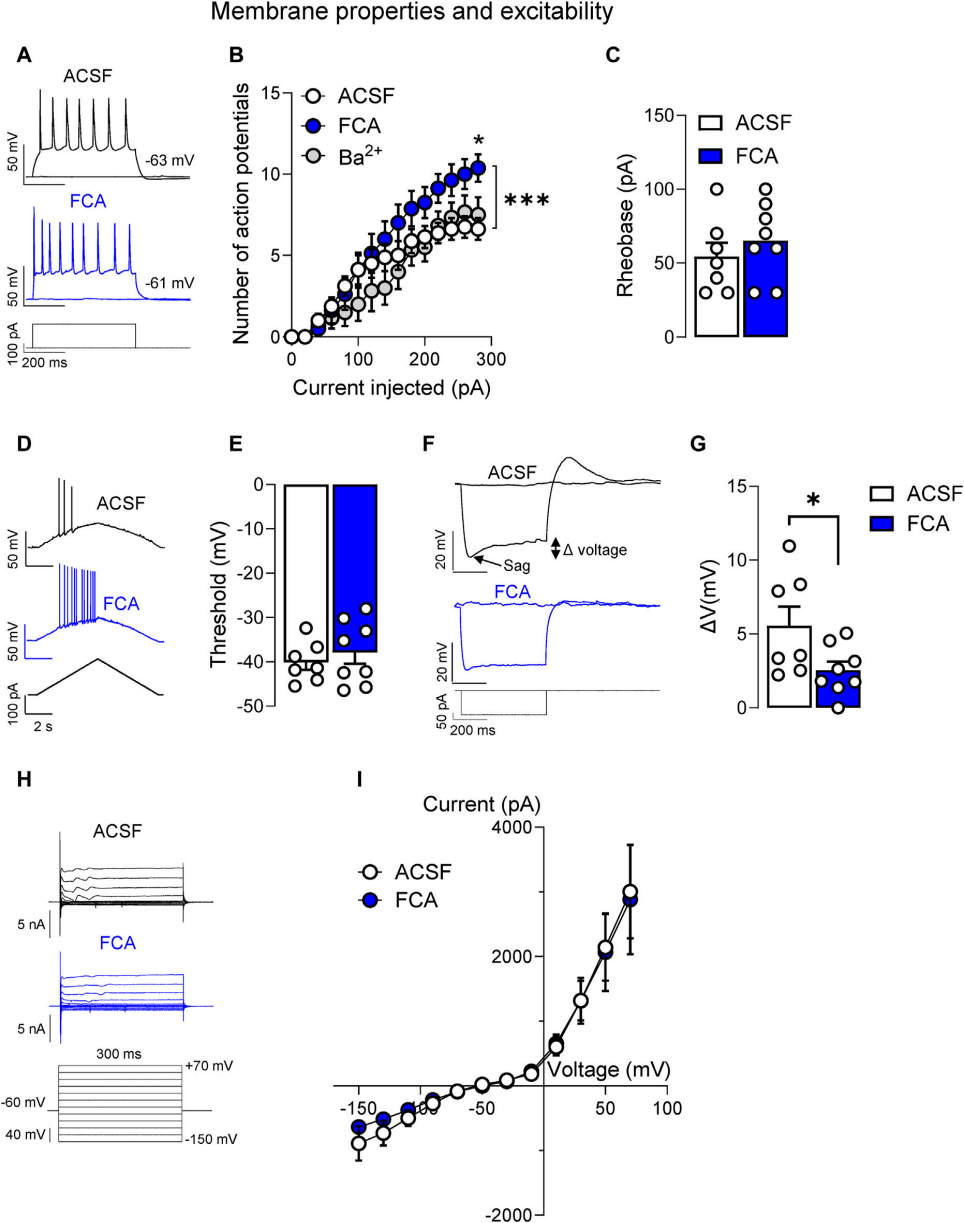
Facilitatory effects of astrocyte inhibition by fluorocitric acid (FCA) on CeLC neuronal properties in a neuropathic model. Neurons recorded from FCA-treated slices (100 μM, 1 h) showed increased neuronal excitability **(A,B)** induced by depolarizing current injections, while Ba^2+^ pretreatment had no effects compared to the control (ACSF) group (*, *p* < 0.05, two-way ANOVA with Bonferroni *post hoc* tests). Symbols show means ± SEM. ***, *p* < 0.001, two-way ANOVA. In the same neurons, FCA had no effects on rheobase **(C)** or AP threshold **(D,E)**, but it decreased the depolarizing voltage sag **(F,G)** without affecting the I-V relationship **(H,I)** compared to the control (ACSF) treated cells. Bar histograms show means ± SEM. *, *p* < 0.05, unpaired student t-tests. Symbols show means ± SEM, two-way ANOVA. ACSF, n = 7; FCA, n = 8.

**FIGURE 3:**
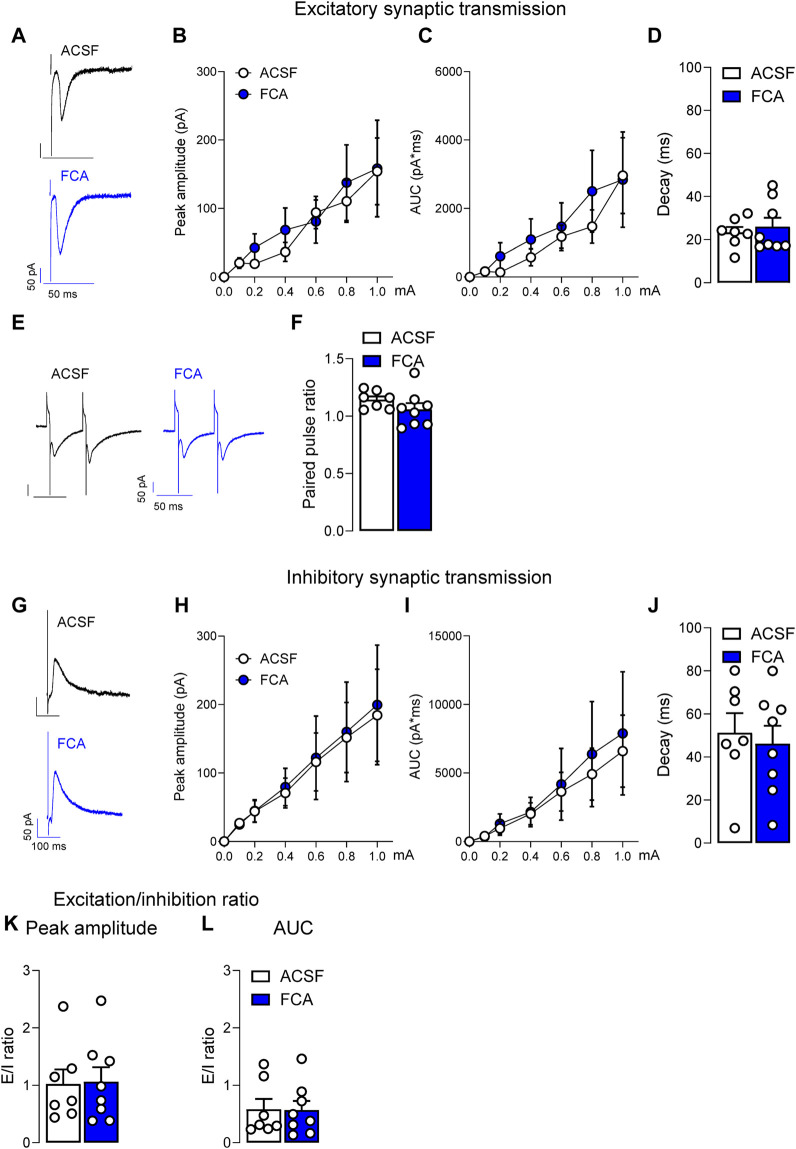
Lack of effects of astrocyte inhibition by FCA on evoked synaptic responses in a neuropathic model. Pre-treatment of brain slices with FCA (100 μM, 1 h) had no effect on the peak amplitude, area under the curve (AUC) or decay time of mono-synaptic excitatory post-synaptic currents (EPSCs) evoked by the electrical stimulation of PB input to CeLC neurons **(A–D)** recorded in brain slices obtained from neuropathic animals. Additionally, no change was observed in the paired-pulse ratio at the PB-CeLC synapse **(E,F)**. Similarly, pre-incubation with FCA (100 μM, 1 h) did not affect the peak amplitude, AUC or decay time of glutamate-driven inhibitory post-synaptic currents (IPSCs) evoked by the electrical stimulation of PB afferents onto CeLC neurons **(G–J)** in the neuropathic pain condition. No significant change was observed in the excitation/inhibition (E/I) ratio **(K,L)**. Symbols show means ± SEM, two-way ANOVA. Bar histograms show means ± SEM, unpaired t-tests. ACSF, n = 7; FCA, n = 8.

Considering that significant changes of GFAP astrocytic marker at mRNA and protein level were found only at the chronic phase of the neuropathic pain model (Figure 1), we focused our analysis of astrocyte functions on the chronic neuropathic stage (4-week time point). Whole-cell patch clamp experiments were performed on non-CRF neurons located in the CeLC to evaluate the effects of fluorocitric acid (FCA) on the neuronal properties (Figure 2) and synaptic responses evoked by the electrical stimulation of PB inputs (Figure 3) based on the results obtained from the immunohistochemical analyses showing increased GFAP (+) staining in both CeC and CeL in chronic SNL rats. Brain slices were incubated with FCA barium salt (100 μM, 1 h) and then transferred to the recording chamber which allowed the washout of Ba^2+^. Neurons in FCA-treated slices showed increased neuronal excitability (Figures 2A, B) (*p* < 0.05, two-way ANOVA with Bonferroni’s *post hoc* tests) but no changes of rheobase (Figure 2C) or AP threshold (Figures 2D, E) compared to the control (ACSF) group. In the same neurons, FCA pretreatment decreased the depolarizing voltage sag (Figures 2F, G) without affecting the I-V relationship (Figures 2H, I) compared to vehicle control (ACSF) group, suggesting that astrocytes may modulate neuronal excitability through mechanisms involving I_h_. In order to avoid confounding results on neuronal excitability due to the presence of Ba^2+^ salt in the FCA solution, some brain slices were incubated with BaCl_2_ (100 μM, 1 h) which resulted in no significant effects compared to control (ACSF) group (Figure 2B) (ACSF, n = 7; FCA, n = 8, Ba^2+^, n = 6; Figure 2B, current injected (row) factor, F_(14, 285)_ = 38.87, *p* < 0.001; treatment (column) factor, F_(2, 285)_ = 18.88, *p* < 0.001; interaction, F_(28, 285)_ = 1.178, *p* = 0.2500; two-way ANOVA; Figure 2C, *p* = 0.4293, t = 0.8158; Figure 2E, *p* = 0.5017, t = 0.6910; Figure 2G, *p* < 0.05, t = 2.198, unpaired t-tests; Figure 2I, voltage (row) factor, F_(11, 156)_ = 27.02, *p* < 0.001; treatment (column) factor, F_(1, 156)_ = 0.1075, *p* = 0.7434; interaction, F_(11, 156)_ = 0.0640; *p* > 0.9999, two-way ANOVA).

In the same neurons, no significant effects of FCA on excitatory or inhibitory synaptic transmission were observed. Peak amplitude, area under the curve (AUC) or decay time of EPSCs (Figures 3A–D) or paired-pulse ratio (PPR; Figures 3E, F) recorded at the PB-CeLC synapse were not significantly different between FCA or ACSF treated groups (ACSF, n = 7; FCA, n = 8; Figure 3B, current (row) factor, F_(6, 91)_ = 5.886, *p* < 0.001; treatment (column) factor, F_(1, 91)_ = 0.3429, *p* = 0.5596; interaction, F_(6, 91)_ = 0.1287, *p* = 0.9924; Figure 3C, current (row) factor, F_(6, 91)_ = 4.860, *p* < 0.001; treatment (column) factor, F_(1, 91)_ = 0.7441, *p* = 0.3906; interaction, F_(6, 91)_ = 0.1737, *p* = 0.9833, two-way ANOVA; Figure 3D, *p* = 0.6040, t = 0.5315; Figure 3F, *p* = 0.2161, t = 1.300, unpaired t-tests). FCA also had no effect on glutamate-driven IPSCs evoked by the electrical stimulation of PB input (Figures 3G–J) onto CeLC neurons, suggesting that astrocytes were not involved in synaptic transmission at the PB-CeLC synapse (ACSF, n = 7; FCA, n = 8; Figure 3H, current (row) factor, F_(6, 91)_ = 4.883, *p* < 0.001; treatment (column) factor, F_(1, 91)_ = 0.0467, *p* = 0.8294; interaction, F_(6, 91)_ = 0.0092, *p* > 0.999; Figure 3I, current (row) factor, F_(6, 91)_ = 3.475, *p* < 0.01; treatment (column) factor, F_(1, 91)_ = 0.2246, *p* = 0.6367; interaction, F_(6, 91)_ = 0.0430, *p* = 0.9997, two-way ANOVA; Figure 3J, *p* = 0.6892, t = 0.4090, unpaired t-tests). No significant change was observed in the excitation/inhibition ratio (Figures 3K, L) (ACSF, n = 7; FCA, n = 8; Figure 3K, *p* = 0.9113, t = 0.1136; Figure 3L, *p* = 0.9573, t = 0.0545, unpaired t-tests).

### 3.3 Behavioral effects of astrocyte inhibition at the chronic stage of neuropathic pain

**FIGURE 4:**
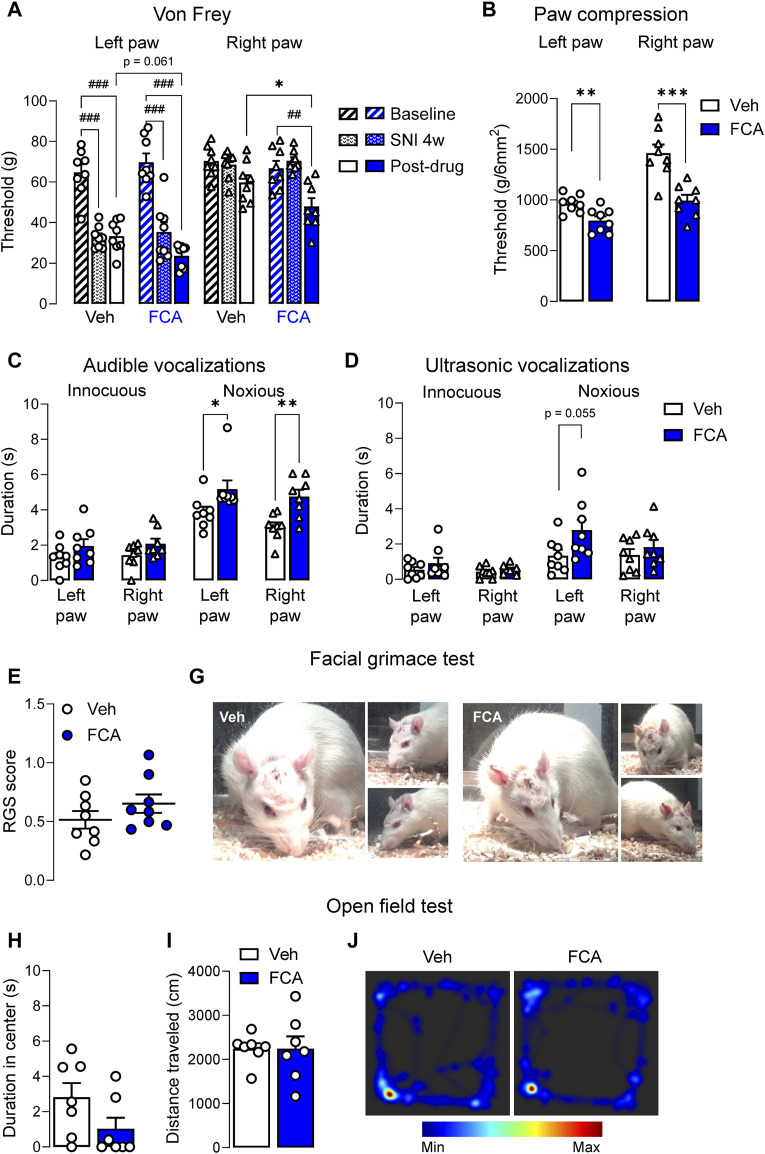
Pronociceptive effects of astrocyte inhibition by FCA in CeA on neuropathic pain-like behaviors. **(A)** Mechanical thresholds (measured by electronic von Frey) of the left (injured), but not right, hind paw were significantly decreased 4 weeks after SNL surgery, confirming the neuropathic pain condition. Stereotaxic injection of FCA (100 μM, 1 μL) into the CeA significantly lowered mechanical withdrawal thresholds on the right hind paw and further decreased the thresholds on the left hind paw compared to vehicle (Veh) treatment. Bar histograms show mean ± SEM. ^##^, ^###^ *p* < 0.01, 0.001 compared to baseline (pre-SNL surgery). *, *p* < 0.05, compared to vehicle, two-way ANOVA with Bonferroni’s multiple comparisons. Intra-CeA administration of FCA (100 μM, 1 μL) significantly decreased the withdrawal thresholds measured by compression **(B)** of the left (injured) and right paws and increased the audible vocalizations **(C)** evoked by noxious, but not innocuous, stimulation of the left (injured) and right hind paws, but had no significant effect on the ultrasonic vocalizations **(D)** though a trend for a facilitatory effect was observed for the noxious compression of the left (injured) hind paw. Injection of FCA (100 μM, 1 μL) into the CeA showed a non-significant trend to facilitatory effects on the facial grimace scale **(E,G)** and anxiety-like behaviors measured in the open field test **(H–J)** compared to vehicle. Bar histograms show mean ± SEM. *, **, ***p < 0.05, 0.01, 0.001 compared to vehicle, unpaired student t-tests. (A-G) Veh, n = 8; FCA, n = 8; (H-L) Veh, n = 7; FCA, n = 7.

To determine the behavioral consequences of astrocyte inhibition in the amygdala, pain-like behaviors were measured after FCA (100 μM, 1 μL) or vehicle injection into the CeA of SNL rats. See 3.2 for the rationale to focus on the chronic stage (4-week time point). SNL surgery significantly increased mechanosensitivity on the left (injured) hind paw in the von Frey assay (Figure 4A) (*p* < 0.001 compared to baseline, two-way ANOVA with Bonferroni’s posthoc tests), confirming the neuropathic pain condition. FCA delivered into the CeA (see 2.7.1) significantly decreased the mechanical withdrawal thresholds on the right hind paw and decreased the thresholds on the left hind paw even further compared to the vehicle-treated group in SNL rats (Figure 4A; *p* < 0.05 compared to vehicle, two-way ANOVA with Bonferroni’s posthoc tests). Significant effects were observed after SNL induction or FCA treatment (Figure 4A, vehicle = 8, FCA = 8, left paw, treatment (column) factor, F_(1, 14)_ = 0.04457, *p* = 0.8358; time (row) factor, F_(2, 28)_ = 79.23, *p* < 0.001; interaction, F_(2, 28)_ = 2.785, *p* = 0.0789; right paw, treatment (column) factor, F_(1, 14)_ = 4.016, *p* = 0.0648, time (row) factor, F_(2, 28)_ = 13.89, *p* < 0.001; interaction, F_(2, 28)_ = 2.166, *p* = 0.1334, two-way ANOVA with Bonferroni’s posthoc tests). FCA administered into the right CeA of neuropathic rats significantly decreased the withdrawal thresholds measured by compression (Figure 4B) of the left (injured) paw (*p* < 0.01, t = 3.176, unpaired t-tests) and right paw (*p* < 0.001, t = 4.571, unpaired t-tests). The data suggest that astrocyte inhibition in the amygdala exacerbates hypersensitivity in chronic pain.

FCA also increased audible vocalizations (Figure 4C) evoked by noxious, but not (normally) innocuous, stimulation of the left (injured) paw (innocuous, *p* = 0.1779, t = 1.419; noxious, *p* < 0.05, t = 2.215, unpaired t-tests) and right paw (innocuous, *p* = 0.1056, t = 1.730; noxious, *p* < 0.01, t = 3.586, unpaired t-tests) paw, but had no effect on ultrasonic vocalizations (Figure 4D, left paw, innocuous, *p* = 0.3049, t = 1.065; noxious, *p* = 0.0550, t = 2.094; right paw, innocuous, *p* = 3,523, t = 0.9622; noxious, *p* = 0.4122, t = 0.8453, unpaired t-tests) though a non-significant facilitatory effect of FCA was observed on ultrasonic vocalizations evoked by noxious compression of the left (injured) hind paw compared to vehicle treatment (vehicle, n = 8; FCA, n = 8). A non-significant trend for differences between the FCA or vehicle treated groups was observed on the facial grimace scale score (Figures 4E, G; vehicle, n = 8; FCA, n = 8; *p* = 0.2260, t = 1.267, unpaired t-tests) and on anxiety-like behaviors measured in the OFT (Figures 4H–J; vehicle, n = 7; FCA, n = 7; Figure 4H, *p* = 0.1061, t = 1.747; Figure 4I, *p* = 0.9992, t = 0.0010, unpaired t-tests). The data suggest that inhibition of astrocytes may affect evoked behaviors (reflexes and vocalizations) rather than spontaneous behaviors (grimace and open field tests).

### 3.4 Validation of astrocyte inhibition by FCA

**FIGURE 5:**
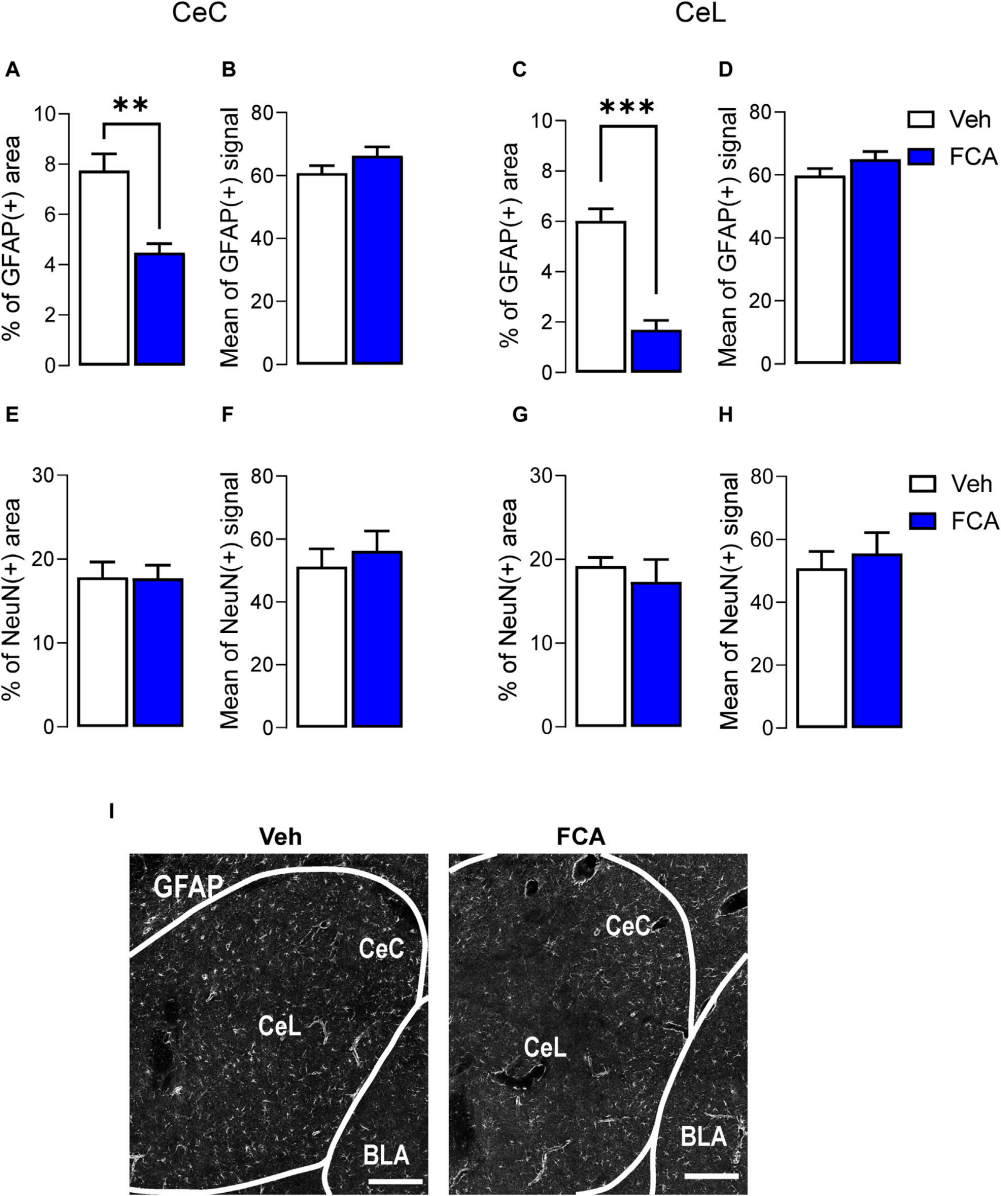
Validation of astrocyte inhibition by FCA in CeA in neuropathic rats. Immunohistochemical analysis showed decreased percentage of astrocytic GFAP positive area, but not mean of positive signal, in the CeC **(A,B)** and CeL **(C,D)** after FCA (100 μM, 1 μL) injection into the CeA of SNL (4 weeks) rats compared to vehicle (Veh) group, consistent with astrocyte inhibition in the targeted area. NeuN staining showed no significant differences between the two groups **(E–H)**, confirming the glia-specific effect of the pharmacological approach. Bar histograms show mean ± SEM. **, ***p < 0.01, 0.001 compared to vehicle, unpaired student t-tests. Veh, n = 5; FCA, n = 5 **(I)** Representative images of GFAP (+) staining in brain sections from Veh (left) and FCA (right) injected neuropathic rats. Scale bar = 200 μm.

Immunohistochemical analysis of GFAP and NeuN staining was used to validate the pharmacological approach. After the behavioral experiments, the animals were perfused and tissue collected for immunohistochemical analysis, which revealed that in the CeC and CeL, the area of GFAP (+) marker (Figures 5A, C), but not the mean of positive signal (Figures 5B, D), was significantly decreased after FCA (100 μM, 1 μL) microinjection compared to vehicle group in chronic SNL rats. Importantly, no differences in NeuN marker signal were observed between the two groups (Figures 5E–H), confirming the selective inhibition of astrocytes rather than neurons in the targeted region (vehicle, n = 5; FCA, n = 5 animals; Figure 5A, *p* < 0.01, t = 4.347; Figure 5B, *p* = 0.1702, t = 1.507; Figure 5C, *p* < 0.001, t = 7.074; Figure 5D, *p* = 0.1581, t = 1.557; Figure 5E, *p* = 0.9566, t = 0.05619; Figure 5F, *p* = 0.5683, t = 0.5949; Figure 5G, *p* = 0.5247, t = 0.6651; Figure 5H, *p* = 0.5931, t = 0.5565, unpaired t-tests). Analysis of GFAP and GS markers in the CeL area at high resolution (×60 oil immersion objective) confirmed the selectivity of the pharmacological approach (Supplementary Figure S1).

## 4 Discussion

This study addressed an important knowledge gap concerning the contribution of astrocyte signaling to amygdala functions in a model of chronic pain. The involvement of neuroimmune elements to pain processing has been shown extensively in preclinical research [for reviews (Tian et al., 2012; Salter and Stevens, 2017; Donnelly et al., 2020; Grace et al., 2021)] and most recently in clinical studies (Loggia et al., 2015; Albrecht et al., 2018; Albrecht et al., 2019; Albrecht et al., 2021), but mechanistic research was largely focused on peripheral and spinal nociceptive processing. Less is known about supraspinal neuroimmune signaling as a pain mechanism, and little if anything about the situation in the amygdala with regard to pain conditions. The key findings of this project are: 1) Increased reactive astrocytes in the amygdala were observed only at the chronic phase (4-week time point) of a neuropathic pain model and 2) selective pharmacological inhibition of the astrocytic population in the amygdala (CeA) had facilitatory electrophysiological and behavioral effects in the chronic pain condition. These results were somewhat unexpected based on existing literature pointing to beneficial effects of interventions aimed to silence glia at peripheral and spinal levels in pain conditions. It should be noted that research has largely focused on the modulation of specific glial factors, while we targeted the astrocytes themselves. Thus, the data suggest differences between amygdala and peripheral and spinal nociception and a unique role of astrocytes.

**FIGURE 6:**
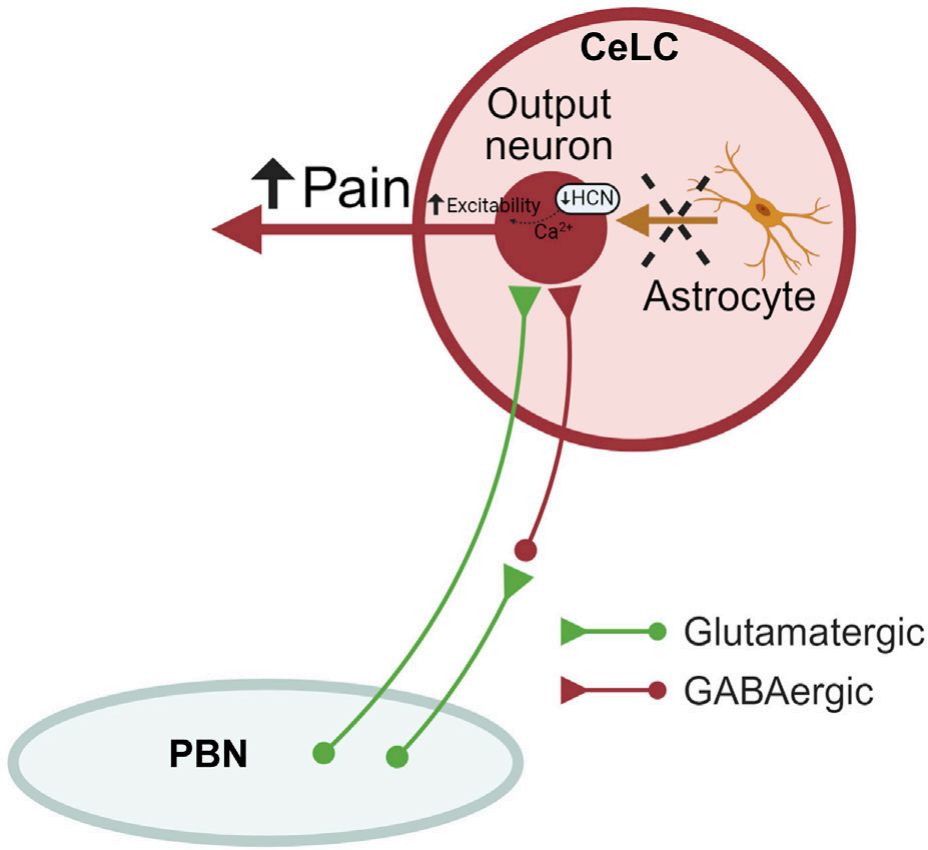
Astrocyte signaling in the amygdala (CeA) in neuropathic pain. Astrocytic silencing increases the output of CeLC neurons through mechanisms involving decreased HCN signaling, resulting in increased neuropathic pain-related behaviors without affecting synaptic transmission at the PB-CeLC synapse. Therefore, endogenous astrocyte signaling may sustain protective neuronal functions. CeLC, laterocapsular division of the central nucleus of amygdala; PBN, parabrachial nucleus; HCN, hyperpolarization-activated cyclic nucleotide–gated (HCN) channels. Created with BioRender.com.

Evidence is as follows. Astrocyte activation was measured as increased GFAP(+) mRNA and protein levels in the CeA at the chronic (4 weeks) but not acute (1 week) stage (Figure 1), pointing to a delayed astrocytic response in the amygdala in neuropathic pain. Surprisingly, in patch-clamp experiments, selective inhibition of astrocyte metabolism achieved with the incubation of the brain slices with FCA enhanced excitability of CeA neurons through a mechanism that involved I_h_ (Figure 2) in the absence of synaptic effects (Figure 3), arguing against generalized non-specific inhibitory effects of FCA treatment. As a consequence, intra-CeA microinjection of FCA resulted in increased evoked vocalizations and decreased mechanical withdrawal thresholds (in the von Frey and paw compression tests), and non-significant facilitatory effects in the grimace test and anxiety-like behaviors (OFT) in rats 4 weeks after SNL induction (Figure 4), supporting the idea that amygdala astrocytes may serve beneficial protective functions in chronic pain (Figure 6). It remains to be determined if these differential behavioral effects reflect a beneficial role of astrocytes on sensory *versus* affective aspects or rather on evoked *versus* spontaneous behaviors. Importantly, GFAP (+) and GS (+), but not NeuN (+), staining decreased after FCA administration into CeA (Figure 5 and Supplementary Material), indicating that FCA selectively affected astrocytes but not neuronal functions in the amygdala.

The amygdala plays a critical role in the development and maintenance of pain and pain modulation. Several lines of research have focused on the modulation on neuronal factors to reduce uncontrolled amygdala activity as a desirable strategy to mitigate pain [for reviews (Thompson and Neugebauer, 2017; Thompson and Neugebauer, 2019; Neugebauer, 2020)]. The contribution of neuroinflammation at different levels of the pain neuroaxis, especially in supraspinal regions, is a more recent area of investigation. A recent study explored the effects of exogenous activation of astrocytes in the amygdala in a neuropathic pain model (Wahis et al., 2021), whereas our study is the first study to show the consequences of inhibition of astrocytic signaling in the amygdala, hence the role of endogenous astrocyte function, in pain processing and pain modulation.

Previous studies demonstrated that intrathecal injections of FCA had antinociceptive effects in zymosan- (Clark et al., 2007) or formalin- (Watkins et al., 1997) induced peripheral inflammatory, neuropathic (SNL) (Cao et al., 2014), and chronic post-ischemia (Tian et al., 2017) pain models. Similarly, intra-RVM FCA decreased carrageenan-induced thermal and mechanical hypersensitivity (Roberts et al., 2009) and ameliorated infraorbital tactile allodynia in a model of trigeminal neuropathy (chronic constriction injury to the unilateral infraorbital nerve) (Wei et al., 2008) in rats. Additionally, repeated injections of FCA into the PAG had inhibitory effects on mechanical allodynia in a model of diabetic neuropathic pain induced by systemic streptozotocin (Liu X. et al., 2022), suggesting that astrocyte activation engaged the descending facilitatory pain system. In contrast, our results point to a beneficial role of amygdala astrocytes in a chronic pain condition. This is more in line with data showing beneficial functions of astrocytes in the forebrain. In a model of focal cerebral ischemia (middle cerebral artery occlusion), repeated injections of FCA into the motor cortex reduced vascular remodeling of the ischemic area and induced neurological deficits in rats (Hayakawa et al., 2010) while intracerebral administration of FCA induced seizures events in anesthetized rats (Willoughby et al., 2003; Broberg et al., 2008).

The dual role of astrocytes in neuropathology is only beginning to emerge, and two different phenotypes of reactive astrocytes have been proposed. The A1 subtype seems to be induced by neuroinflammation and promotes cells death, while the A2 subtype, induced by ischemia, promotes tissue healing and repair (Liddelow et al., 2017). Reactive astrocytes have been associated with several disorders in preclinical research, including pain (Li et al., 2019), although the astrocytic subtype involved (A1 vs. A2) remains to be determined. Importantly, A1 astrocytes have been implicated in neurodegenerative disorders such as Alzheimer’s, Parkinson’s and Huntington’s disease, in clinical studies (Liddelow et al., 2017). If and how reactive astrocytes contribute to chronic pain mechanisms is not clear yet, perhaps because glial cells undergo a series of morphological and molecular changes related to time, region, microenvironment, and type of insult. The relative contribution of A1 and A2 subtypes to the findings of the present study remains to be determined. Additionally, the role of amygdala astrocytes in pain mechanisms is an open question considering that pain-behaviors and neuroplasticity persist in chronic pain condition, suggesting that astrocyte activation, even though “beneficial”, is not sufficient to accomplish pain relief, but perhaps other non-neuronal elements such as microglia or oligodendrocytes drive maladaptive pain plasticity.

In support of the idea of a protective role of astrocytes in amygdala pain mechanisms, a recent study found that bilateral pharmacological or optogenetic activation of a subpopulation of astrocytes containing oxytocin receptors decreased anxiety-like behaviors in a neuropathic pain model whereas mechanical hypersensitivity was improved only by the pharmacological treatment; this effect was linked to increased inhibition of output neurons in the medial CeA, but it is unclear if mechanistic analyses were performed in pain model. Although this study did not explore effects of astrocyte inhibition and did not account for hemispheric lateralization (Wahis et al., 2021), the results with exogenous activation of astrocytes complement our findings with inhibition of endogenous astrocyte signaling. Preclinical [for reviews (Thompson and Neugebauer, 2017; Neugebauer, 2020; Allen et al., 2021)] and most recently clinical evidence (Barroso et al., 2023) points to preferential pain-related lateralization to the right amygdala. The complexity of the neuroimmune system and the intricate bidirectional interactions between neuronal and non-neuronal cells pose challenges to the better understanding of the chronic pain processing and more effective therapeutic options available in the clinics.

Our electrophysiological data suggest inhibitory effects of astrocyte function on neuronal hyperexcitability in chronic neuropathic pain. Under physiological conditions, astrocytes sustain important neuronal functions throughout the central nervous system, including regulation of energy and blood flow, control of extracellular levels of K^+^ or neurotransmitters (glutamate and GABA), and support of synaptic properties (Han et al., 2021; Valles et al., 2023). Astrocytes have been extensively implicated in inhibitory neurotransmission (especially GABA-A receptors) and K^+^ or I_h_ currents (Bellot-Saez et al., 2017; Lezmy et al., 2021; Liu et al., 2021; Liu J. et al., 2022). We found effects of FCA on excitability and Ih currents, but not synaptic transmission, suggesting region- and model-specific functions. As a note of caution, the presence of Ba^2+^, a well-known K^+^ channel blocker (Rohaim et al., 2020), in the FCA solution used for incubation of the brain slices before recordings started, did not appear to confound the patch-clamp data, because FCA had significantly different effects on neuronal excitability compared to brain slices pre-treated with BaCl_2_ (Figure 2B). Additionally, recordings were done in brain slices superfused with ACSF after incubation in FCA Ba^2+^ salt, and Ba^2+^ is known to wash out quickly (Sutor and Hablitz, 1993; Shi et al., 2000; Xiao et al., 2011). Accordingly, evidence from a previous study established that the effects of FCA observed on neuronal functions were not determined by residual Ba^2+^ in the bath (Wenker et al., 2010). The impairment of hyperpolarization-activated cyclic nucleotide-gated (HCN) cation channels could explain the observed effects of FCA on the depolarizing voltage sag, although this was not confirmed by pharmacological blockade in our experiments. Importantly, Ba^2+^ should not interfere with I_h_ (Ludwig et al., 1998). The lack of effects on synaptic transmission at the PB-CeLC synapse could be explained by the fact that amygdala neuroplasticity may not be sustained by synaptic plasticity in this pathway at the chronic pain stage.

Some limitations of this work should be considered. Although a strong sexual dimorphic influence of the neuroimmune system on pain-related mechanisms has been observed and represents an important line of research (Rosen et al., 2017; Presto et al., 2022), this study does not address sex specific differences in amygdala astrocyte function in pain. Moreover, the involvement of astrocytic signaling in amygdala pain functions was determined at the chronic but not acute stage of neuropathic pain, based on molecular and immunohistochemical evidence for changes in astrocyte activation at the chronic but not acute neuropathic pain stage (Figure 1). The identity of reactive astrocyte subtypes and the influence of astrocytic signaling on specific neuronal ion channels besides I_h_ currents also remain to be investigated. The current study provides the rationale for future studies into the role of astrocyte function in different brain regions and different stages of different pain models.

## Data Availability

The raw data supporting the conclusions of this article will be made available by the authors, without undue reservation.
