# Supplementary material for: Impaired amygdala astrocytic signaling worsens neuropathic pain-associated neuronal functions and behaviors
Source: Front Pharmacol. 2024 Mar 21;15:1368634. doi: 10.3389/fphar.2024.1368634 (PMC10991799; doi:10.3389/fphar.2024.1368634)
Supplement: Supplementary file 1 [file DataSheet1.pdf]

Supplementary Material

1. Methods

For general information about immunohistochemical procedures refer to section 2.5 in main text. Primary antibodies used: Chicken-anti-GFAP (Novus NBP1-05198, 1:1000); Rabbit-anti-GS (GeneTex, #GTX109121) (1:250); Guinea Pig-Anti-NeuN (Millipore) ABN90, 1:1000); Secondary antibodies used (1:1000): Goat-anti-Chicken Alexa488 (A11039); Goat-anti-Guinea Pig Alexa 647 (A21450), Goat-anti-Rabbit Alexa-568 (A11011). Sections were imaged at identical conditions on confocal microscope (Olympus FV3000), using 3×3 mosaic, Z = 6 taken with 60× oil immersion objective, in three channels: GFAP, GS and NeuN. Image analysis was performed with ImageJ software (ImageJ v1.53f51, NIH) as described in the methods (see 2.5).

Supplementary Figure 1

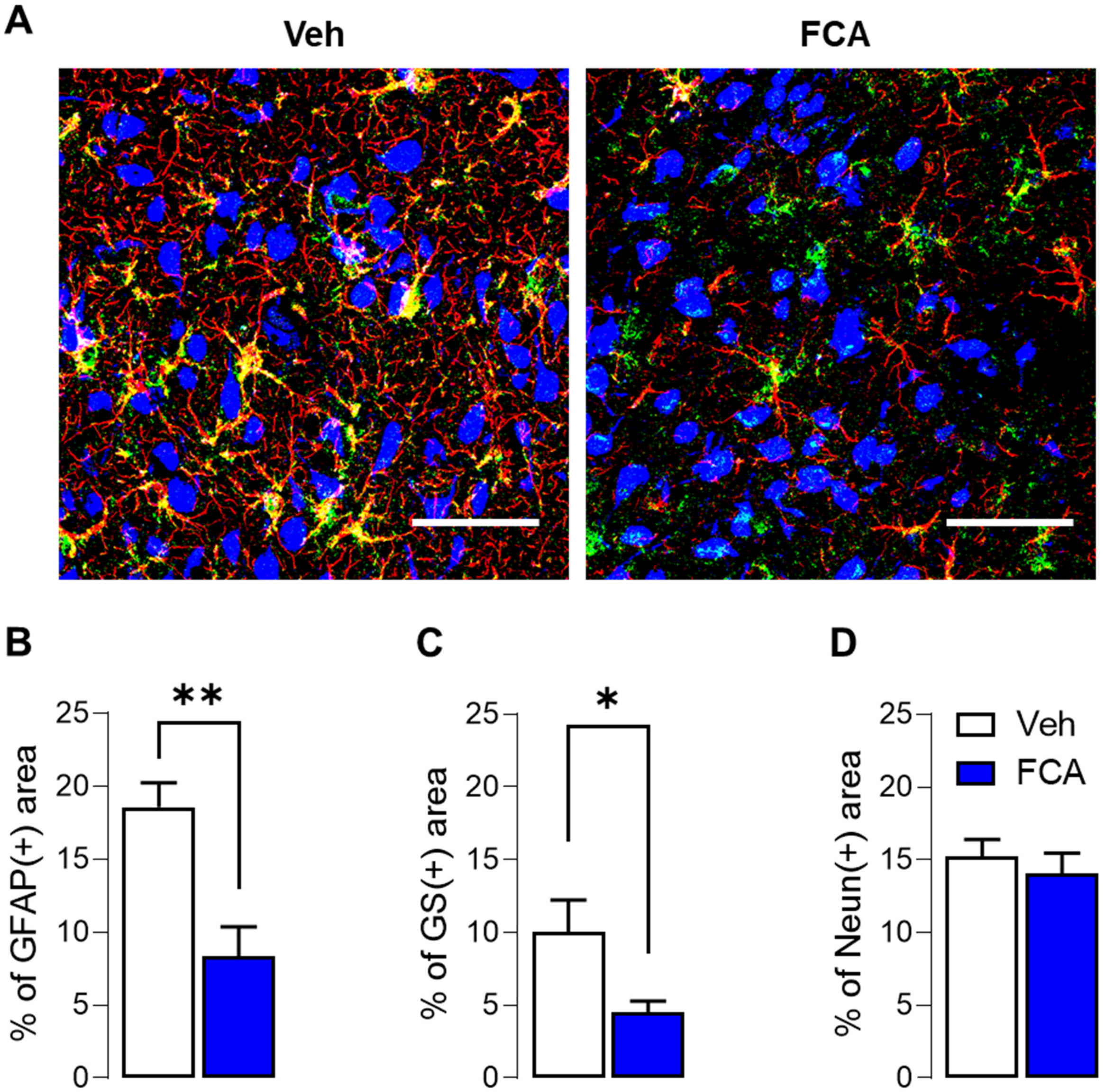

Supplementary Figure 1. FCA-induced specific astrocytic signaling reduction in CeA. (**A**) Representative images of GFAP(+) (red), GS(+) (green) and NeuN(+) (blue) staining in CeL area from Veh (left) and FCA (right) injected neuropathic rats. Scale bar = 50 µm. Immunohistochemical analysis showed decreased GFAP(+) (**B**) and GS(+) (**C**), but not NeuN(+) (**D**), signal in the CeL of FCA-treated animals compared to the control (Veh) group, suggesting that FCA-related effects were specific to astrocytes. Bar histograms show mean ± SEM. *, ** $p < 0.05$, 0.01 compared to vehicle, unpaired student t-tests. (B) $p < 0.01$, $t = 3.925$; (C) $p < 0.05$, $t = 2.378$; (D) $p = 0.5386$, $t = 0.6424$; vehicle, $n = 5$; FCA, $n = 5$ animals. GFAP = Glial fibrillary acidic protein; GS = Glutamine Synthetase; NeuN = Neuronal Nuclear Antigen.
